# Nutritional Interventions in Osteoarthritis: Mechanisms, Clinical Evidence, and Translational Opportunities

**DOI:** 10.3390/nu18020244

**Published:** 2026-01-13

**Authors:** Milan Patel, Gabriela Betanzos, Marco Troka, Jay Modi, George Nageeb, Alan D. Kaye, Alaa Abd-Elsayed

**Affiliations:** 1 New York Institute of Technology College of Osteopathic Medicine, Arkansas State University, Jonesboro, AR 72401, USA; mpatel0503@gmail.com; 2 Department of Neuroscience and Behavior, University of Notre Dame, Notre Dame, IN 46556, USA; 3 Department of Anesthesiology, School of Medicine and Public Health, University of Wisconsin, Madison, WI 53726, USA; mtroka@wisc.edu; 4 New York Institute of Technology College of Osteopathic Medicine, Old Westbury, NY 11545, USA; modijay12003@gmail.com; 5 School of Medicine, Stanford University, Stanford, CA 94305, USA; 6 Department of Anesthesiology, Louisiana State University Health Sciences Center, Shreveport, LA 71103, USA; alan.kaye@lsuhs.edu

**Keywords:** osteoarthritis, cartilage, chondrocytes, oxidative stress, inflammatory, inflammation, curcumin, polyphenols, nanoparticles, nutrition, herbal

## Abstract

Osteoarthritis (OA) is a leading cause of chronic pain worldwide. This is driven by progressive cartilage degradation, inflammation, oxidative stress, and metabolic dysfunction. Current pharmacologic interventions mostly lead to symptomatic relief without actually affecting disease progression. Thus, there is a growing interest in the development of new interventional methods. Our review seeks to synthesize preclinical, translational, and clinical evidence on the impact nutritional methods have on OA management. Whole-diet approaches, such as Mediterranean and plant-based, have been linked to reduced pain, increased physical function, and positive biomarker changes. Bioactive compounds, including curcumin, polyphenols, omega-3 fatty acids, and select herbal extracts, have shown anti-inflammatory, antioxidant, and chondroprotective effects via NF-κB, Nrf2, AMPK, and SIRT1 pathways. This review particularly focuses on plant-derived substances. Emerging nanoparticle technology with regard to advanced delivery systems shows initial promise in nutraceutical pharmacokinetics and tissue targeting. Overall, nutritional interventions are adjunct interventions to OA management. Although these are not full treatment replacements, dietary modifications and targeted nutraceutical strategies with improved delivery systems may lead to more preventive, personalized, and holistic OA management and care.

## 1. Introduction

Osteoarthritis (OA) affects a growing and expansive portion of the population worldwide. The current estimates indicate that over half a billion people are living with OA worldwide, a disease marked by joint pain, stiffness, and progressive disability [1]. Between 1990 and 2020, OA prevalence increased by over 100%, and the years lived with disability (YLDs) have also risen sharply, creating a substantial economic burden on patients and healthcare systems [1]. Middle-aged and older adults are disproportionately affected, and OA has been designated a “serious disease” by the FDA, mainly related to its chronic impact on quality of life [1]. Risk factors such as age, obesity, prior joint injury, and genetics drive this alarming upward trend, especially obesity, as it accounts for a growing fraction of the OA burden worldwide. In this regard, the joints most commonly affected by OA include the knee, hand, and hip [1].

Modern medical treatments for OA primarily focus on symptom relief rather than a cure. Analgesics and non-steroidal anti-inflammatory drugs (NSAIDs) can ease patient pain, and intra-articular injections of steroids or hyaluronic acid may provide short-term relief. Still, none can entirely stop the disease’s progression [2]. Long-term use of NSAIDs or corticosteroids carries gastrointestinal and cardiovascular risk and often fails to protect cartilage [3]. Ultimately, many patients require joint replacement surgery, which is not only costly but also usually only available at advanced stages and carries its own limitations, including implant longevity and revision needs. Currently, no pharmacological or surgical therapy reliably restores damaged cartilage or prevents OA progression.

Given these limitations, there is a growing interest in nutrition-based strategies for the prevention and management of OA. Diet and lifestyle are factors that can be modified and influence various OA pathways, including body weight, systemic inflammation, and metabolic health. For example, diets rich in anti-inflammatory omega-3 fatty acids have been shown to reduce systemic inflammation, alleviate joint pain, and improve function in patients with OA [4]. On the other hand, Western diets high in omega-6 fatty acids can exacerbate synovitis and cartilage breakdown. Emerging evidence also links gut microbiome and micronutrient status to OA outcomes, suggesting that food components may affect disease processes at the molecular level [5]. The novel nutritional interventions may serve both preventive and disease-modifying roles by targeting risk factors, such as obesity, and molecular mechanisms that underlie OA. It is important to note that nutritional interventions serve as an adjunct therapy rather than a complete replacement.

This narrative review, therefore, aims to integrate dietary, molecular, and therapeutic perspectives to provide a comprehensive understanding of how nutrition can influence the onset and progression of OA. We start by outlining the underlying pathophysiological mechanisms that drive cartilage degeneration and joint inflammation, establishing the biological rationale for nutrition-based modulation. We then examine whole-diet patterns, specific bioactive plant compounds, and isolated natural molecules, including their mechanistic pathways and therapeutic potential. This review will particularly focus on plant-derived substances and their effects on cartilage, rather than on all joint tissues affected by OA. To address the persistent challenge of poor bioavailability, we explore new and emerging nanoparticle and advanced delivery technologies designed to enhance the clinical utility of nutraceuticals. The review concludes with an assessment of translational and clinical evidence, safety considerations, and future directions. These sections culminate in clarifying how nutritional interventions, supported by modern molecular insights and delivery innovations, may contribute to more effective, holistic, and personalized strategies for preventing and managing osteoarthritis.

Importantly, OA is now recognized as a whole-joint disease rather than an isolated disorder of articular cartilage. With articular cartilage, the initial changes can be found at the joint surfaces where mechanical stresses are the greatest [6]. In addition to cartilage degeneration and synovial inflammation, structural and metabolic alterations of subchondral bone, bone marrow lesions, menisci, ligaments, and periarticular adipose tissue contribute to disease progression and pain. The variable degrees of synovial inflammation coupled with degeneration of ligaments and menisci contribute to hypertrophy of the knee joint capsule [7]. Subchondral bone sclerosis, bone marrow edema-like lesions, and aberrant bone remodeling are strongly associated with pain severity and structural progression, suggesting potential mechanistic links between systemic metabolism, nutrition, and OA outcomes [8].

This manuscript is a narrative review that synthesizes preclinical, translational, and clinical literature on nutritional interventions in OA. Literature was identified through searches of PubMed, Scopus, and Web of Science, focusing primarily on studies published between approximately 2000 and 2025. Priority was given to systematic reviews, meta-analyses, randomized controlled trials, and mechanistic studies relevant to OA pathophysiology, dietary patterns, nutraceuticals, and emerging delivery systems. Given the narrative nature of the review, no formal systematic selection or risk-of-bias assessment was performed.

## 2. Pathophysiology of Osteoarthritis and the Role of Inflammation

OA is a multifactorial joint disease characterized by degenerative and inflammatory processes that can lead to joint degradation and deterioration. As understanding of OA increases, evidence suggests that chronic low-grade inflammation is a primary contributor to disease progression [9,10]. The pathophysiology of this inflammation involves many associations within the joint and systemic inflammatory contributors. From a biomechanical perspective, chondrocytes play a crucial role. In the initial stages of OA, chondrocytes are able to repair the matrix with no positive result. However, the leakage of proteoglycans and type II collagen breakdown is induced. Furthermore, water concentration increases, implying there has been a reduction in tensile strength of the ECM [11]. A summary of the physiologic components mentioned throughout this section will be presented in summary Table 1 at the conclusion of this section.

### 2.1. Cartilage Degradation and Chondrocyte Dysfunction

Articular cartilage within the joint consists of a specialized cell type, known as a chondrocyte, which produces an extracellular matrix (ECM), enabling smooth and fluid movement. These cells are often dormant but are driven to a pro-inflammatory state, leading to progressive damage and a primary contribution to OA [7]. Chondrocytes are capable of both producing and responding to inflammatory cytokines and chemokines during ECM degradation [12]. OA cartilage and OA chondrocytes are particularly sensitive and can produce the primary inflammatory cytokines TNF, IL-1β, and IL-6 [13]. Matrix metalloproteinases (MMPs, especially MMP-13) and aggrecanases (ADAMTS-4, ADAMTS-5) are amplified by these pro-inflammatory cytokines, contributing to the structural support of cartilage, including type II collagen and aggrecan [14,15].

### 2.2. Synovial Inflammation

Synovial inflammation is often indicative of the presence and progression of OA [16,17]. Within this tissue, synovial macrophages exist that are a key contributor to pro-inflammatory molecules and interactions with chondrocytes, including cytokines, MMPs, and tissue inhibitor metalloproteinases [18]. M1 macrophages accelerate cartilage breakdown by releasing cytokines and degradative enzymes, whereas M2 macrophages support tissue repair [18]. A greater ratio of M1/M2 balance is correlated with OA severity [18].

A key component of knee OA is the Infrapatellar Fat Pad (IFP), also known by the name Hoffa’s Fat Pad. The IFP receives a vast amount of blood supply from the surrounding synovial membrane and thus plays a critical role in recovery of surrounding structures [19,20]. In terms of OA pathogenesis, in OA individuals, the IFP in knee OA patients was displayed with increased inflammatory infiltration, vascularization, and thickness of interlobular septa. Furthermore, in comparison to subcutaneous adipose tissue (SAT), the IFP showed increased fibrosis and vascularization with greater lymphocytic infiltration of the interlobular septum and smaller fat lobules [19,20,21]. Thus, these findings are indicative of IFP playing a deleterious role in knee OA.

### 2.3. Oxidative Stress and Mitochondrial Dysfunction

Mitochondria play a critical role in mediating cellular stress through their relationship with apoptosis. Dysfunction of the mitochondria in OA chondrocytes is characterized by the production of reactive oxygen species (ROS), impaired electron transport chain activity, and loss of mitochondrial membrane potential [22]. This impact can further ECM degradation and chondrocyte apoptosis [22,23]. Ultimately, these factors further promote MMPs and aggrecanases, ultimately increasing the contribution to OA [22,23].

### 2.4. Systemic Inflammatory Contributors

Obesity and metabolic syndrome increase the mechanical load on the joint; however, these conditions highlight the evidence of systemic inflammation contributing to OA [24]. Adipose tissue releases the previously discussed pro-inflammatory cytokines (TNF, IL-1β, and IL-6) and adipokines, which have a heightened effect on chondrocyte catabolism and cartilage damage [24,25]. TNF-α, IL-1, and IL-6 are elevated in the synovial fluid, membrane, and cartilage of OA patients, with a higher presence of synovial inflammation in patients with obesity [25,26]. Patients with knee OA have been shown to have significant associations with the occurrence and progression of OA with several components of metabolic syndrome, including higher systolic blood pressure, lower serum HDL cholesterol levels, and higher body mass index [27].

### 2.5. Pro-Inflammatory Diets

Dietary factors, particularly diets high in saturated fat, are correlated with increased levels of circulating free fatty acids in plasma [28]. OA cartilage demonstrates the accumulation of palmitate and oleate, the primary saturated and monounsaturated fatty acids in human tissue [29]. Such increased levels of free fatty acids are correlated with the severity of cartilage lesions consistent with OA [27]. Elevated plasma lipids have also been found, consistent with high-fat diets, within the previously discussed cartilage catabolism, which is associated with the upregulation of MMP-13, autotaxin signaling, COX-2, and cytokines IL-6 and TNF-α [30,31,32].

## 3. Dietary Patterns and Whole-Food Approaches

Dietary patterns are playing a growing role in understanding the progression and symptom burden of osteoarthritis [33]. A summary of the key dietary patterns and diets mentioned in this section are presented in summary Table 2 at the conclusion of this section. Key diets that have shown correlation to the development of osteoarthritis include the Mediterranean diet, Western diet, and plant-based diets. Each plan offers a distinct nutritional profile and potential influence on inflammation, cartilage health, and symptom progression. The Mediterranean diet is notable for incorporating foods such as fruits, vegetables, legumes, white meat, dairy products, and olive oil, highlighting its efforts to reduce the consumption of red meat and processed meat in consumers’ diets [34]. As a result of these modifications, the Mediterranean diet is rich in monounsaturated fats, polyphenols, and omega-3 fatty acids. These components are essential in the pathophysiology of osteoarthritis, which is mediated by the prolongation of pro-inflammatory cytokines such as IL-1, IL-6, and TNF-α [35]. These cytokines modulate chondrocytes, which produce cartilage, ultimately increasing nitric oxide (NO) and prostaglandin E2, both of which are involved in pain associated with osteoarthritis. Studies have shown that the monounsaturated fats in the Mediterranean diet, such as those found in olive oil, contain polyphenols that provide anti-inflammatory activity by decreasing the synthesis of cytokines, nitric oxide, and prostaglandins [35]. Another component of the Mediterranean diet is fish, which offers polyunsaturated fats (PUFAs), including omega-3 fatty acids.

As a result, compounds, such as resolvins, docastrienes, etc., derived from omega-3 fatty acids result in decreased gene expression of cartilage proteinases and inflammatory cytokines that contribute to osteoarthritis symptoms [35]. A study in the UK has quantified this pathophysiologic mechanism of OA by measuring sCOMP, a marker of cartilage degradation, as well as various other biomarkers [36]. Results indicate that subjects’ post-Mediterranean diet consumption showed a decrease in IL-1α and sCOMP, demonstrating a potential benefit for osteoarthritis patients. Additionally, functional measurements were assessed through hip and knee range of motion, where significant improvements were observed in the Mediterranean diet group [36]. Additionally, the Mediterranean diet has been shown to improve osteoarthritic pain. A 2019 cross-sectional study analyzing adherence to the Mediterranean diet showed significant outcomes in knee pain, as measured by the WOMAC pain scale [37]. The mechanism behind the decrease in knee pain was attributed to the reduction of pro-inflammatory cytokines, such as IL-1α, that the Mediterranean diet provides. Overall, the Mediterranean diet appears to improve OA outcomes by reducing pro-inflammatory cytokines and oxidative stress, limiting cartilage degradation; however, a strong and statistically significant connection has not emerged. Yet, these results are promising and still warrant further research, as this provides cautious optimism. Another diet known for its association with exacerbating OA symptoms is the Western diet. This diet is characterized by excessive eating and snacking, supplementing a Western lifestyle that is often filled with physical inactivity and insufficient sleep [38]. This high-fat diet, along with Western alcohol consumption, has shown impairment to the gut-mucosal layer, causing increased lipopolysaccharides (LPS) in circulation [39,40]. Recent mouse studies have found that displaced LPS from the bacterial membrane initiates obesity and ultimately activates an immune response through systemic inflammation, exacerbating osteoarthritic symptoms. This mainly works by activating the TLR4 pathway, which is a mediator of inflammation in osteoarthritis [39,40]. Alongside this, dyslipidemia resulting from the Western diet promotes LDL cholesterol synthesis, which has been shown to increase the production of pro-inflammatory mediators [41]. At a cellular level, the Western diet has been associated with obesity biomarkers, including leptin and resistin. Leptin is a peptide hormone that circulates in those with higher body fat. Recent studies have shown that leptin is positively correlated with its concentration in synovial fluid and the severity of OA [41].

Additionally, leptin has been shown to increase the catabolism of articular cartilage related to increased gene expression of matrix metalloproteinases (MMPs), which break down cartilage [41]. Additionally, another biomarker, resistin, is also correlated with insulin resistance, commonly associated with obesity. Furthermore, increased resistin levels have been detected in the synovial fluid of osteoarthritic patients, showing a positive correlation with radiographic early-stage knee OA [41]. Overall, the biomarkers leptin and resistin were elevated in the experimental group of knee OA as compared to the controls. Thus, these biomarkers, in conjunction with additional lab parameters and monitoring devices, could be used for determining disease progression and intervention [41].

The Western diet, which is typically high in fat content, has been linked to increased risk of OA development. The excess dietary fat stored in adipose tissue, muscle, and liver creates an inflammatory state within the body. Studies in mice have found that the rate of OA is increased in those provided a high-fat diet. From this study, it was observed that OA progression was increased as a result of decreased cartilage thickness and increased osteophyte diameter, which is related to the metabolic dysregulation induced by a high-fat diet [42]. The underlying mechanism of this is a result of inflammatory cytokines being released, leading to metabolic dysfunction that exacerbates cartilage breakdown through the activation of MMPs and overall oxidative stress.

Ultimately, plant-based diets have been associated with a reduction in OA symptoms. The prevalence of plant-based foods has surged as a result of efforts to decrease society’s carbon footprint and increased awareness of the harmful effects of greenhouse gases [43]. The plant-based diet removes animal meat from the consumer’s diet entirely and replaces it with tempeh, quinoa, and lentils. This diet provides lower levels of saturated fats and an increase in fiber and phytochemicals, which can help lower LDL cholesterol [43]. Dietary approaches target a greater aspect of the individual’s dietary consumption, while isolated compounds create a change on a smaller level. Thus, a dietary approach change leads to greater effectiveness in positive cases than isolated compounds. A specific type of phytochemical is polyphenols, which are metabolites produced by almost every fruit and vegetable. Polyphenols provide the ability to inhibit reactive oxygen species (ROS) and ultimately reduce expression of pro-inflammatory genes that are activated in OA [44]. Thus, consuming foods high in these nutrients allows for a decrease in the production of ROS that ultimately destroys chondrocytes in cartilage. To support this idea, a 2022 study analyzed whether a diet higher in phytochemicals, as found in plant-based foods, would affect the odds of developing knee OA [45]. Amirkhizi et al. found that men in the highest tertile of the dietary phytochemical index (DPI) were 76% less likely to have knee OA as compared to those in the lowest tertile. Furthermore, women were 58% less likely to have knee OA when comparing the highest and lowest tertiles [45]. This demonstrates that adherence to a phytochemical-rich diet, as calculated by a dietary phytochemical index, was associated with lower odds of knee OA. Thus, the prevalence of plant-based diets rich in phytochemicals has a positive effect on OA.

Another aspect of the plant-based diet is the abundance of fiber and antioxidant-rich foods. Fiber is found in various components of the plant-based diet, such as fruits and grains, aiding in the digestion of food from the small to the large intestine [46]. Additionally, dietary fiber has been shown to activate the NF-κB pathway, reducing pro-inflammatory metabolites and overall inflammation. Alongside fiber, antioxidants are a viable strategy to minimize osteoarthritis inflammation. In the damaged joints of osteoarthritis, increased inflammatory factors cause oxidative stress, which contributes to cartilage degradation [47]. As a result, mitigating these pro-oxidant factors through antioxidant consumption in a plant-based diet can be a potential treatment for patients. Such compounds can be found in abundance in herbs and fruits, which are commonly found in a plant-based diet.

## 4. Bioactive Natural Compounds and Plant Extracts

### 4.1. Plant Extracts

Given the fundamental impact that inflammation has on the pathophysiology of OA, it is essential to gain a comprehensive understanding of disease management. There are emerging benefits of several bioactive natural compounds and plant extracts that may contribute to the advancement of OA management.

### 4.2. Curcumin

Curcumin is the primary active compound in turmeric (*Curcuma longa*) and is isolated from the dried South Asian plant’s rhizome. Curcumin has been extensively investigated for its ability to inhibit NF-κB phosphorylation, demonstrating efficacy as an anti-inflammatory and anti-cytokine agent [48,49,50]. An in vitro study of articular chondrocytes demonstrated that curcumin exhibited suppressing effects on NF-κB activation, contributing to the inhibition of COX-2 and MMP-9 expression [49]. Curcumin treatment also suppressed IL-1β-induced NF-κB activation by inhibiting both IκBα phosphorylation and degradation [51]. This mechanism revealed the anti-inflammatory properties of curcumin, which allowed for catabolic mediation in signaling pathways in chondrocytes [51]. In addition, curcumin was further shown to downregulate the activation of pro-inflammatory NF-κB proteins while simultaneously upregulating SOX-9 [48]. Together, these results provide evidence for the protective effects of curcumin on cartilage autophagy and anti-inflammatory properties [48,49,50,51].

#### 4.2.1. Boswellia Serrata

Boswellia serrata gum resin is an oleo-gum resin native to India and the Middle East and exists in six distinct boswellic acid forms. Specifically, keto-β-boswellic acid (KBA) and 3-O-acetyl-11-keto-β-boswellic acid (AKBA) have been studied for their notable anti-inflammatory effects in suppressing T cells and for significant symptom improvement in OA patients in clinical applications [52,53,54]. The mechanism of boswellia was recently explored in an in vivo animal model, where OA-induced mice treated with boswellia serrata gum resin extract downregulated several key OA markers, including COX-2, IL-6, TNF-α, MMP-3, and MMP-13 [54]. These serve as the basis for some level of inhibition of inflammatory effects in cartilage matrix destruction, namely through suppressing MMP-3 and MMP-13 [55]. Suppression of these OA hallmarks indicates a potential for anti-catabolic effects and improved OA management [55].

#### 4.2.2. Green Tea Catechins

(-)-Epigallocatechin 3-gallate (EGCG) is a bioactive polyphenol located within green tea and has demonstrated evidence for anti-inflammatory effects [55,56,57]. This occurs through the EGCG-induced inhibition of nitric oxide synthase in human chondrocytes stimulated with IL-1β, resulting in the disruption of NF-κB activation [58]. Further ECGC studies with IL-1β in human cartilage have also shown inhibition of MMP-1 and MMP-13 [57]. In addition to reduced MMP-13 and COX-2 expression in OA cartilage after ECGC treatment, recent research also reveals a decrease in chondrocyte apoptosis [57]. This enhanced chondroprotection demonstrated a reduction in arthritic changes in animal models through enhanced autophagy function and a reduction in the inflammatory pathway [59]. The added element of increased autophagy serves as a protective factor against OA, reducing cartilage degradation [59].

#### 4.2.3. Gingerols

Gingerols are the primary phenolic compounds in many forms of widely popularized ginger (*Zingiber officinale*). Ginger has been used extensively for centuries due to its anti-inflammatory applications [60,61]. 10-Gingerol emerged as the most potent of the three gingerols due to the promotion of enhanced antioxidant gene expression and NRF2 nuclear translocation [62]. This modulation of oxidative stress was attributed to the ability of 10-gingerol to activate the KEAP1-NRF2-ARE axis and inhibit NF-κB signals [62]. Modulation of apoptotic regulation and pro-inflammatory cytokines is significant for increased cartilage regeneration and ECM balance, which is vital in OA management [62].

#### 4.2.4. Resveratrol

Resveratrol is a polyphenol found in the skin of red grapes and has been demonstrated to have antioxidant and anti-inflammatory properties [63,64]. In vivo and in vitro research on temporomandibular joint OA investigates the antioxidant and protective role of resveratrol in modulating and inhibiting COX-2 and NF-κB expression [64]. Furthermore, the results find significant antioxidant effects against chondrocyte apoptosis as a result of inhibition of COX-2 and NF-κB expression [64]. An additional animal study found further chondrocyte maintenance in OA through decreased ER stress markers and inflammatory cytokines, including TNF-α, IL-1β, and MMP-13, by utilizing resveratrol [65].

### 4.3. Herbal Formulations and Traditional Medicine

The application of these bioactive natural compounds and plant extracts is deeply rooted in Ayurvedic, Chinese, and Mediterranean herbal combinations. Such combinations may enable additive and/or synergistic anti-inflammatory and analgesic effects, as demonstrated by the interaction of multiple signaling pathways [66,67]. While research has provided significant evidence for the anti-inflammatory properties of these herbal formulations, there are limitations within this research. Given the interaction of these pathways, it may be challenging to isolate a mechanistic understanding [68]. Standardization of these compounds remains a significant limitation; differences in origins of harvest and cultivation may lead to discrepancies between products [68]. The opportunity for heterogeneity in these compounds may pose a health risk either from contamination or from cheap or poorly distilled extracts [67]. This raises questions about risk factors, including the stability of these products given varying production environments [69]. This lends itself to investigating the bioavailability of these compounds. Additionally, the availability of cartilage through oral ingestion has been questioned, given the relatively low vascular nature of articular cartilage [69].

## 5. Isolated Natural Molecules and Synthetic Analogs

There has been increasing attention towards isolated natural molecules and synthetic analogs for targeted OA treatments. These compounds are able to allow for greater, precise, and mechanistic investigation. Furthermore, improved standardization and potentially enhanced bioavailability are added benefits as compared to the traditional herbal options. Many of these molecules are able to exert their effects through modulation of key inflammatory, oxidative, and catabolic pathways involved in OA pathogenesis.

### 5.1. Polyphenols and Flavonoids

Quercetin and Kaempferol are classified as flavonoids that fall within the larger class of bioactive molecules called polyphenols, which have been previously discussed for their anti-inflammatory and antioxidant properties [47]. Quercetin, a flavonoid commonly found in fruits and herbs, has recently demonstrated the ability to inhibit apoptosis in chondrocytes and possesses anti-inflammatory properties [70]. The promotion of polarization to M2 macrophages, associated with tissue repair, was also observed [70]. Further research into local and systemic pro-inflammatory mechanisms in OA via quercetin was conducted in animal studies. Quercetin was shown to inhibit MMP-3 and MMP-13 while promoting the expression of aggrecan and collagen II [70]. A reduction in inflammatory cytokines in synovial fluid and serum, as well as protection of subchondral trabecular bone, was also attributed to high-dose quercetin [70]. Kaempferol, a flavonoid also found in fruits and vegetables, has been shown to exhibit anti-inflammatory and antioxidant effects [71]. Kaempferol was used in an animal study to demonstrate significant inhibition of (IL)-1β-induced expression of the common COX-2 and MMP-1,3,13 [72]. Kaempferol was also shown to inhibit the mitogen-activated protein kinase (MAPK) pathway [72].

### 5.2. Terpenes

Terpenes are a separate classification of molecules that have gained traction in recent years for their involvement in cannabis therapy for the management of disease symptoms. Among terpenes, β-caryophyllene (BCP) and β-myrcene have been studied for their potential in vivo efficacy in anti-inflammatory properties [73]. An investigation into the mechanistic pathways in a study utilizing hemp seed oil and terpenes (BCP and β-Myrcene) demonstrated the synergistic effects of IL-6 pathway induction and simultaneous reduction in IL-1α [74]. This contributes to a systematic review of the analysis of terpenes’ ability to regulate pro-inflammatory mediators in animal models [75].

Furthermore, BCP is also able to decrease oxidative stress in chondrocytes. However, when BCP is used in conjunction with ascorbic acid (AA) and d-glucosamine (GlcN), BCP can have synergistic effects on these inflamed chondrocytes. A study conducted by Mattiuzzo et al. showed that with the addition of 1 µM of BCP, reduced IL-1β expression of CM-treated cells at 6 h after administration. Consequently, in combination with AA and GlcN, this further reduced IL-1β in addition to NF-κB1 at 6 and 12 h after administration (*p* < 0.05, *p* < 0.01) [76]. The ability of synergistic effects to be displayed when using BCP, for example, with AA and GlcN, continues to further progress the viability of different OA treatment options.

### 5.3. Carotenoids

Carotenoids are abundant in orange, yellow, or red pigmented fruits and vegetables. With more than 750 natural forms, carotenoids have been discovered to possess antioxidant and anti-inflammatory properties [77]. An emerging cross-sectional study using the National Health and Nutrition Examination Survey found that higher trans-lycopene serum levels are associated with a negative correlation to OA [78]. This was clearer with increasing age [78]. The phytochemical crocin also possesses anti-inflammatory and therapeutic effects for OA. In a study conducted by Lei et al., OA rats were administered 30 mg/kg for 10 days. It was found that after treatment, joint pain, IL-6 levels, muscular lipid peroxidation (MLO), and Nrf2 levels were decreased. On the other hand, citrate synthase, MHC IIa expression, glutathione production, and glutathione peroxidase activity were increased [79,80]. In another study, this one by Ding et al., crocin was found to repress IL-1β expression and reduce expression of MMP-1,-3, and -13 in chondrocytes [79,81]. These studies showed that crocin exhibits therapeutic effects in OA cases through alleviating oxidative stress and inflammation, as well as reducing cartilage degeneration.

### 5.4. Additional Studies

As discussed in Section 4.2, many natural herbal formulations have limited bioavailability, which depends on the route of administration, harvesting site, and environmental conditions, thereby limiting standardization and stability. To address limitations, research has emerged on synthetic analogs and their potential to enhance bioavailability in OA. A chemically modified curcumin analog was tested in OA animal models and found a similar reduction in the NF-κB/HIF-2α axis and MMP-3 expression. Furthermore, modulation of chondrocyte apoptosis was observed [82]. Another study utilized Next Generation Ultrasol Curcumin in OA rats, a form that is 64.7 times more bioavailable than natural 95% curcumin, with significant inflammatory mediation [83]. Lipid-core nanoformulated resveratrol was found to have a substantial impact in reducing NO levels in human chondrocytes [84,85]. Omega-3 analogs, eicosapentaenoic acid (EPA) and docosahexaenoic acid (DHA), are a final area of emerging interest in OA, oftentimes in supplement form. Investigation into the role of omega-3 polyunsaturated fatty acids revealed a chondroprotective effect in the modulation of MMP-3 and COX-2, providing a possible benefit to OA patients [84].

## 6. Nanoparticles and Advanced Delivery Systems

Many nutraceuticals and bioactive plant-derived compounds relevant to OA, including curcumin, resveratrol, polyphenols, and omega-3 lipids, are limited by poor oral bioavailability. These compounds are often lipophilic, rapidly metabolized, and subject to extensive first-pass clearance, resulting in low and variable concentrations reaching joint tissues following ingestion [86]. Such pharmacokinetic constraints have stimulated interest in advanced delivery systems designed to enhance stability, tissue penetration, and local bioavailability.

Nanoparticle-based delivery platforms have emerged as a promising strategy to address these limitations. Importantly, however, the vast majority of nanoparticle-enabled nutraceutical approaches for OA remain at the preclinical or early translational stage, with evidence derived primarily from in vitro systems and animal models. At present, human data are sparse, and these technologies should be viewed as experimental adjuncts rather than established clinical interventions.

### 6.1. Common Nanoparticle Formulations in OA Research

A variety of nanoscale carriers have been investigated for OA-related applications, including lipid-based nanoparticles (liposomes and solid lipid nanoparticles), biodegradable polymeric nanoparticles (PLGA, chitosan, and hyaluronic acid-based systems), polymeric micelles, and injectable hydrogels. These platforms offer several theoretical advantages, such as improved aqueous solubility of hydrophobic compounds, protection from enzymatic degradation, prolonged time residing within the joint space, and controlled or sustained drug release.

Lipid-based nanoparticles are among the most extensively studied systems due to their biocompatibility and ability to encapsulate both hydrophilic and lipophilic agents. Preclinical studies have demonstrated that lipid-encapsulated curcumin or resveratrol achieves higher intra-articular concentrations and longer half-life compared with free compounds [87]. Similarly, polymeric nanoparticles fabricated with chitosan or PLGA can be engineered for tunable degradation and sustained release and may be functionalized with cartilage-binding ligands or hyaluronic acid to enhance joint retention.

### 6.2. Preclinical Evidence and Mechanistic Rationale

In animal models of OA, nanoparticle-mediated delivery of phytochemicals has consistently demonstrated superior anti-inflammatory and chondroprotective effects relative to non-encapsulated compounds. For example, curcumin-loaded chitosan or hyaluronic acid-coated nanoparticles have been shown to suppress NF-κB signaling, reduce expression of matrix-degrading enzymes such as MMP-1 and MMP-13, and preserve type II collagen content in cartilage [88,89]. Comparable findings have been reported for nanoformulated resveratrol and other antioxidant compounds, which reduce nitric oxide production, oxidative stress, and inflammatory cytokine release in joint tissues.

Beyond direct effects on chondrocytes, nanoparticle delivery systems may also influence synovial inflammation and immune cell polarization. Preclinical studies suggest that targeted intra-articular nanoparticle delivery can reduce synovitis and shift macrophage populations toward anti-inflammatory phenotypes, thereby attenuating local cytokine production [90]. These findings support a mechanistic rationale for improved efficacy through localized, sustained delivery rather than systemic exposure.

### 6.3. Translational Limitations and Clinical Relevance

Despite encouraging preclinical results, significant translational barriers limit the current clinical applicability of nanoparticle-based nutraceutical delivery in OA. These include heterogeneity in nanoparticle composition and manufacturing methods, challenges in large-scale production and standardization, regulatory complexity, and limited long-term safety data for repeated intra-articular or systemic exposure. Moreover, differences in joint size, biomechanics, and immune responses between animal models and human models complicate extrapolation of efficacy.

At present, few nanoparticle-based nutraceutical formulations have progressed to well-designed human trials in OA, and none are incorporated into clinical guidelines. Consequently, while advanced delivery systems represent an important area of innovation with potential to overcome bioavailability constraints, their role in OA management remains investigational. Future research should prioritize rigorously designed translational studies that integrate pharmacokinetic assessment, safety evaluation, and clinically meaningful outcomes to determine whether these technologies can meaningfully augment nutritional interventions in human osteoarthritis.

## 7. Mechanistic Insights into Nutritional Modulation of Inflammatory Pathways

When understanding the mechanism behind osteoarthritis pathology, it is essential to note the nutritional modulation of inflammatory pathways. High levels of reactive oxygen species (ROS) are produced in osteoarthritic cartilage. As a result, inflammatory cytokines are secreted, which produce nitric oxide (NO), another pro-inflammatory molecule [44]. This molecule causes the explicit activation of the nuclear factor kappa B (NF-κB) pathway, which increases the pro-inflammatory markers IL-1β and TNF-α. It is important to note that there are several plant-derived antioxidant compounds that have been shown to attenuate ROS accumulation and inhibit NF-κB activation. Therefore, this reduces downstream inflammatory cytokine production in osteoarthritic cartilage. To counteract the increased inflammatory and ROS production, the body’s antioxidant defense system activates to remove these molecules [44] efficiently. The system includes peroxiredoxins, glutathione peroxidases, and catalases, all overseen by the nuclear factor (erythroid-derived 2)-like 2 (Nrf2), a transcription factor regulator. It has been shown that in osteoarthritis, this system is dysregulated, resulting in an abundance of ROS, leading to a pro-inflammatory effect. Studies utilizing deferoxamine, an iron chelator, have been used to inhibit iron deposition that occurs in osteoarthritis progression [91]. Iron accumulation exacerbates oxidative stress through redox reactions as well as restoration of Nrf2 signaling. Nrf2 signaling is an important target for minimizing oxidative cartilage damage. Several dietary polyphenols have demonstrated this ability to activate Nrf2 antioxidant pathways, which could serve as a complement to pharmacologic interventions. This study found that deferoxamine contributes to decreasing iron deposition and restoration of the Nrf2 antioxidant system, a crucial pathway for chondrocyte protection.

Additional key inflammatory pathways in osteoarthritic cartilage pathogenesis are the AMPK and SIRT1 pathways. AMPK is a serine/threonine kinase that acts as a regulatory molecule for energy homeostasis [92]. Phosphorylation of this pathway occurs typically in healthy cartilage but is significantly decreased in knee osteoarthritis. As a result, proliferation of inflammatory cytokines occurs, in which the need for pharmacological activators for this pathway could aid in reversing these effects [2]. From a nutritional standpoint, it has been seen that compounds in turmeric, specifically curcumin, have shown the ability to activate the AMPK pathway [2]. The compound can target ADK, which participates in AMPK activation and ultimately dissipates osteoarthritic oxidative stress. This provides a mechanistic link between dietary bioactive compounds and restoration of metabolic signaling pathways that are affected by osteoarthritis. Additionally, the SIRT1 pathway is also a regulatory pathway for energy homeostasis. Similar to AMPK, SIRT1 is decreased in knee OA and leads to an increased apoptotic response, as indicated by biomarkers IL-1β and TNFα [2]. SIRT1 also promotes cartilage-specific gene expression that is chondrocyte protective, making it a key target pathway for osteoarthritis. A common polyphenol in fruits and vegetables known as Fisetin has been linked to decreasing inflammation in osteoarthritis through the SIRT1 pathway [93]. Through SIRT1 activation, fisetin is able to suppress pro-inflammatory signaling and promote cartilage-specific gene expression that is protective against degradation. Fisetin was shown to inhibit IL-1β-induced inflammation by activating the SIRT1 pathway, ultimately decreasing cartilage degradation. Therefore, the AMPK and SIRT1 pathways are key mechanisms for modulating OA inflammation through the use of nutritional compounds. Overall, osteoarthritis has exhibited prominent signs of inflammation in various articular joints, resulting in discomfort and pain for those affected. To modulate such inflammation, it is essential to explore treatments that involve dietary modifications and additions. Our gut microbiome can be a key target for this treatment, and compounds such as oligofructose can become treatment options. As a prebiotic fiber, oligofructose modulates gut microbial composition and reduces systemic inflammatory signaling that can contribute to joint inflammation. Oligofructose has been shown in studies to restore gut microbiomes, acting as a prebiotic fiber and reducing systemic inflammation in the colon and knee, thereby protecting against OA [94]. Thus, the implementation of dietary treatments has been shown to improve inflammatory symptoms associated with osteoarthritis. Targeting molecular pathways, the compounds in foods can provide a safe and accessible way to treat patients from various demographics and geographical locations.

## 8. Translational and Clinical Evidence

Multiple meta-analyses and RCTs have evaluated dietary and nutraceutical interventions in OA, consistently demonstrating modest but statistically significant improvements in pain and function. A 2020 meta-analysis of 42 RCTs found that nutritional supplements, such as glucosamine, curcumin, and collagen, significantly reduced WOMAC pain (standardized mean difference [SMD] ≈ −0.36) and stiffness (SMD ≈ −0.47) compared to the placebo group [95]. Similarly, a systematic review of 69 trials reported that many supplements, like certain polyphenols, Boswellia, and collagen hydrolysate, produced moderate short-term pain relief, with overall moderate effects on pain/function. However, the evidence quality was judged as “very low” due to heterogeneity, small sample sizes, and risk of bias [88].

Curcumin and turmeric extracts have relatively consistently shown improvements in knee pain and function, comparable to those of low-dose NSAIDs [96]. These benefits were seen in VAS pain and WOMAC scores. In diet-focused trials, a meta-analysis of RCTs, conducted through dietary restriction (Mediterranean, plant-based diets, and others), also found significant pain reduction (pooled SMD ≈ −0.65) and functional gains [97]. For instance, a 6-week trial showed that a whole-foods, plant-based diet significantly improved VAS pain and SF-36 physical function compared to control groups [97]. Both isolated nutraceuticals and holistic dietary interventions generally yield statistically significant improvements in pain and function, although their effect sizes tend to be modest.

Post-treatment, some trials also report changes in inflammatory biomarkers. For example, a crossover RCT in overweight adults found that glucosamine plus chondroitin significantly lowered CRP by ~23% compared to the placebo (*p* = 0.048) [98]. In a knee OA RCT, a standardized polyphenol blend, such as curcumin or resveratrol, led to a significant decline in CRP in the treatment group compared to an increase in the vitamin C control group and prevented the rise in IL-6 that was observed in the controls [99]. Other trials of supplements, including fish oil, ginger, and probiotics, have measured CRP/IL-6 with mixed results: some show modest reductions, while others find no significant changes. Overall, nutraceuticals can modulate systemic inflammation biomarkers (CRP, IL-6, TNF-α) to some degree, but these effects are generally small and variable across studies [99].

Comparing whole-food interventions with isolated compounds, evidence suggests that both approaches can be beneficial, but through different mechanisms. Dietary interventions, such as weight-loss or Mediterranean/plant-based diets, often improve OA symptoms, likely by reducing mechanical load and systemic inflammation through weight loss and the synergy of nutrients. The recent diet meta-analysis found that calorie-restricted and plant-based diets significantly improved pain (pooled SMD ≈ −0.65) and physical function (SMD ≈ −0.52) [97]. Similarly, the plant-based diet RCT demonstrated statistically significant, generally modest improvements in patient-reported function and vitality [97]. In contrast, trials of isolated nutrients, such as curcumin, fish oil, vitamins, and botanical extracts, target specific pathways like COX inhibition and cytokine blockade, showing benefit. For example, omega-3 trials report minor improvements in pain, and an RCT of a polyphenol supplement significantly reduced both pain and CRP [99]. Direct head-to-head comparisons of diet versus supplement in the same trial are scarce; however, whole diets inherently address multiple factors related to weight and various macronutrients, whereas supplements focus on single agents.

Key challenges temper these findings. The clinical studies exhibit substantial heterogeneity in terms of design, interventions, and outcomes. Meta-analyses routinely report high *I*^2^ values (~54–66% for pain/function outcomes), due to varied protocols and patient populations [97]. Dosing and formulation vary widely: curcumin trials have used doses ranging from a few hundred to several thousand milligrams per day in different extract forms, making comparisons difficult [96]. Blinding can be problematic in diet trials, and participant adherence is also difficult to monitor; thus, dietary RCTs often rely on self-report and run a high risk of bias. Many nutraceutical trials are short-term, lasting weeks to months; the benefit usually diminishes in longer follow-up circumstances. In fact, the supplement review found no clinically significant effects on pain/function at medium- or long-term follow-ups [88]. Lastly, the overall evidence quality is mixed: most RCTs are small, and systematic reviews have noted generally “low” to “very low” confidence in effect estimates [88]. While translational promise is clear, results must be interpreted with caution related to these methodological challenges.

While many dietary and nutraceutical interventions demonstrate statistically significant improvements in pain and function, only whole-diet interventions consistently achieve effects exceeding established MCID thresholds. Nutraceuticals such as curcumin and glucosamine may reach MCID in select trials or subgroups, but average effects across studies are modest and frequently fall below clinically meaningful thresholds, supporting their role as adjunctive rather than stand-alone therapies.

## 9. Limitations, Safety, and Future Perspectives

### 9.1. Methodological and Practical Limitations

Despite an expanding evidence base linking diet, nutraceuticals, and OA, several limitations constrain the strength and generalizability of current findings. Randomized dietary trials in OA remain relatively few, typically small (encompassing tens to low hundreds of participants), short (6–12 months), and focused predominantly on knee OA in overweight, high-income populations. In the largest recent meta-analysis of randomized dietary interventions (nine RCTs, *n* = 898), dietary change produced moderate improvements in pain and physical function, but with substantial statistical heterogeneity (*I*^2^~60–70%) and considerable variation in diet composition, intensity, and co-interventions, especially exercise and weight-loss support [95].

Heterogeneity is even more pronounced across whole-diet studies. Diets labeled “Mediterranean,” “anti-inflammatory,” “plant-based,” or “low-fat” differ in absolute and relative intakes of fats, carbohydrates, and key phytochemicals, as well as in whether energy restriction is a primary goal. Observational data suggest that prudent and Mediterranean-style patterns, higher total fiber intake, and lower dietary inflammatory index scores are associated with slower symptomatic progression. In contrast, Western-style patterns are associated with worsening pain and function [100,101]. However, residual confounding (e.g., physical activity, socioeconomic status), reverse causality (e.g., diet changes after symptom onset), and self-reported dietary assessment limit the ability to make causal inferences. Nutraceutical trials present additional challenges. The 2020 systematic review of nutraceutical supplements in OA (41 RCTs) showed overall improvements in pain and stiffness (standardized mean differences generally in the small to moderate range), but minimal or inconsistent effects on physical function, and significant heterogeneity across active agents, doses, comparators, and duration [2]. Many trials are single-center, underpowered for structural endpoints, and at moderate risk of bias, with frequent industry sponsorship and incomplete reporting of allocation concealment, adherence, and concomitant therapies.

A major translational limitation is the variability in phytochemical content and bioavailability of plant-derived products. Concentrations of curcuminoids, boswellic acids, catechins, and other bioactives vary with cultivar, growing conditions, harvesting methods, extraction methods, and storage conditions. Even when the same botanical is used, commercial preparations differ in standardization (e.g., percentage of curcuminoids or AKBA), the use of absorption enhancers (e.g., piperine, phospholipids), and particle size (e.g., nanoparticles vs. crude powder). This variability undermines dose–response interpretation and complicates attempts to pool data across trials, as highlighted in recent mechanistic and clinical reviews of nutraceuticals for OA [5,66]. Adherence is another underappreciated limitation. Most dietary studies rely on self-reported food diaries or recalls, which are prone to recall and social desirability bias. Objective biomarkers (e.g., plasma fatty acids, metabolomic signatures of polyphenol intake) are rarely incorporated, and few studies report adherence trajectories over time. Likewise, nutraceutical adherence is often assessed only via pill counts, with limited information on long-term persistence in real-world settings where OA patients typically take multiple medications.

### 9.2. Safety and Toxicity Considerations

From a safety perspective, most clinical trials of dietary changes and common nutraceuticals in OA report adverse event rates similar to those of the placebo, with mild gastrointestinal symptoms or headaches being the most common [2,66]. However, trial populations are typically healthier and younger than the broader OA population, follow-up rarely extends beyond 6–12 months, and rare serious events are unlikely to be captured. Post-marketing pharmacovigilance and case series highlight that specific high-dose botanical preparations may carry a non-trivial hepatotoxic risk. An expanding body of data from the U.S. Drug-Induced Liver Injury Network and other cohorts documents acute hepatocellular injury linked to turmeric/curcumin supplements, with a latency of 1–4 months and characteristic HLA associations [102]. Population-based survey data from the United States indicate that nearly 5% of adults report exposure to one of six botanicals considered potentially hepatotoxic, with turmeric and green tea products being used most frequently [103]. Clinicians can monitor acute liver damage (ALD) in their patients using the revised electronic casualty assessment method (RECAM) to screen for harmful changes [104]. Broader reviews of herbal and dietary supplement hepatotoxicity underscore the importance of meticulous clinical history-taking, rigorous product quality control, and systematic reporting of suspected cases.

These uncertainties are reflected in major rheumatology guidelines. The 2019 American College of Rheumatology/Arthritis Foundation guideline does not recommend routine use of most oral supplements (e.g., glucosamine, chondroitin, vitamin D, fish oil) for knee, hip, or hand OA, mainly because of modest and inconsistent efficacy rather than clear safety signals, while strongly endorsing weight management, exercise, and standard pharmacologic therapies as core treatments [105]. This position underscores an important message: at present, diet and nutraceuticals should be viewed as adjunctive, not replacement, strategies within comprehensive OA care [106,107,108].

Looking forward, several priorities emerge. First, future trials should utilize rigorously characterized and standardized nutritional interventions, with transparent reporting of composition (including macronutrients, key micronutrients, polyphenols, and fatty acids), sourcing, and manufacturing. Second, the incorporation of objective adherence and exposure markers—such as metabolomic fingerprints, circulating fatty acids, and gut microbial or fecal metabolite profiles—will be essential to move beyond self-report and to link specific molecular changes to clinical outcomes. Third, multi-omics and microbiome-informed stratification offer a route toward precision nutrition, identifying subsets of patients who are more likely to benefit from particular dietary patterns or supplements. Finally, nanotechnology and advanced delivery systems may help overcome the pharmacokinetic limitations of key nutraceuticals. Preclinical and early translational work on curcumin nanocarriers, polymeric nanoparticles, and hydrogels suggests improved joint retention, controlled release, and enhanced anti-inflammatory effects in arthritis models, although human data remain limited.

## 10. Conclusions

Taken together, the evidence reviewed here supports nutrition as a biologically plausible and clinically relevant lever in osteoarthritis (OA), but one that is best understood as a disease-modifying adjunct rather than a replacement for established therapies. OA emerges from the convergence of local cartilage catabolism, synovial and systemic low-grade inflammation, oxidative and mitochondrial stress, and metabolic dysregulation. The pathways highlighted in this review, including NF-κB and Nrf2 signaling, AMPK and SIRT1 activity, adipokine and cytokine networks, and the gut–joint axis, are all sensitive to dietary inputs and specific bioactive compounds.

Current evidence, while methodologically heterogeneous, supports the potential for dietary modulation to attenuate inflammatory and degenerative processes. Mediterranean and plant-forward patterns, rich in fiber, unsaturated fats, and phytochemicals, are associated with more favorable symptom profiles and a lower inflammatory burden than Western, high-fat dietary patterns. Randomized trials and meta-analyses of nutritional interventions, particularly energy-restricted and whole-food plant-based or Mediterranean-style diets, demonstrate modest to moderate improvements in pain and physical function, complementing weight-loss–mediated reductions in joint load. Likewise, meta-analyses of nutraceuticals, such as glucosamine, chondroitin, collagen peptides, omega-3 fatty acids, and polyphenol-rich extracts, reveal small but clinically relevant benefits for pain and stiffness in the short term. However, the effects on structure and long-term outcomes remain uncertain.

In the future, optimizing outcomes will require interdisciplinary studies that explicitly link nutrition, molecular biology, and clinical rheumatology. Priorities include standardized, mechanistically informed dietary and supplement protocols; incorporation of imaging, biomarker, microbiome, and multi-omics readouts; and evaluation of advanced delivery platforms (such as nanoparticle-based systems) that can overcome bioavailability barriers for key phytochemicals. The integration of these approaches with guideline-directed exercise and pharmacologic care, as well as emerging AI-enabled tools for precision nutrition and decision support, offers a realistic path toward a more preventive and personalized OA paradigm in which nutrition is not peripheral but central to disease management.

## Figures and Tables

**Table 1:** Summary of OA Pathophysiology and Inflammatory Contributors.

Pathophysiologic Component	Key Cells/Tissues	Primary Mediators	Mechanistic Effects	OA Progression Contribution
Chondrocyte dysfunction and cartilage degradation	Chondrocytes, articular cartilage ECM	TNF, IL-1β, IL-6, MMP-13, ADAMTS-4/5	Increased ECM breakdown (type II collagen, aggrecan), proteoglycan leakage, Increased water content, decreased tensile strength	Structural cartilage loss and impaired repair capacity
Synovial inflammation	Synovial membrane, macrophages (M1/M2)	Pro-inflammatory cytokines, MMPs, TIMPs	M1 macrophages promote catabolism, M2 macrophages support repair, elevated M1/M2 ratio	Accelerated cartilage degradation and disease severity
Infrapatellar Fat Pad (Hoffa’s Fat Pad)	IFP, synovial vasculature, immune cells	Inflammatory infiltrates, fibrotic mediators	Increased Vascularization, fibrosis, lymphocytic infiltration, altered adipose structure	Local inflammatory amplification in knee OA
Oxidative stress and mitochondrial dysfunction	Chondrocyte mitochondria	Reactive oxygen species (ROS)	Impaired ETC activity, loss of mitochondrial membrane potential, apoptosis	Increased MMPs/aggrecanases and progressive ECM degradation
Systemic inflammation (obesity and metabolic syndrome)	Adipose tissue, synovium, cartilage	TNF-α, IL-1β, IL-6, adipokines	Increased Chondrocyte catabolism, increased synovial inflammation, metabolic dysregulation	Increased OA risk, severity, and progression
Pro-inflammatory dietary patterns	Cartilage, plasma lipid pools	Palmitate, oleate, COX-2; IL-6, TNF-α, autotaxin	Lipid accumulation in cartilage, increased MMP-13 and inflammatory signaling	Worsened cartilage lesions and inflammatory burden

**Table 2:** Summary of Dietary Patterns and Effects on OA.

Dietary Pattern	Key Dietary Components	Primary Biological Mechanisms	Key Biomarkers Affected	Effect on OA Outcomes
Mediterranean Diet	Fruits, vegetables, legumes, fish, white meat, dairy, olive oil; low red and processed meat intake	Decreased Pro-inflammatory cytokines (IL-1, IL-6, TNF-α); Decreased nitric oxide (NO) and prostaglandin E2; Decreased cartilage proteinase gene expression; Decreased oxidative stress	↓ IL-1α; ↓ sCOMP (cartilage degradation marker)	Reduced pain (WOMAC score); improved hip and knee range of motion; decreased cartilage degradation; improved inflammatory profile
Western Diet	High saturated fat intake; processed foods; excess calories; alcohol; associated with inactivity and poor sleep	Gut barrier dysfunction → increased circulating LPS; TLR4 pathway activation; Increased systemic inflammation; Increased adipokines (leptin, resistin); increased MMP expression; Increased oxidative stress	↑ Leptin; ↑ Resistin; ↑ LDL cholesterol; ↑ inflammatory mediators	Accelerated OA progression; reduced cartilage thickness; increased osteophyte formation; worsened metabolic dysfunction and inflammation
Plant-Based Diet	Fruits, vegetables, whole grains, legumes (e.g., lentils, quinoa, tempeh); absence of animal meat	Decreased ROS production; Decreased pro-inflammatory gene expression; Decreased LDL cholesterol; antioxidant and phytochemical-mediated cartilage protection; fiber-mediated inflammatory modulation	↓ ROS; ↓ pro-inflammatory cytokines; lower dietary phytochemical index associated with ↓ OA risk	Reduced odds of knee OA; decreased inflammation; potential chondroprotection; improved metabolic and oxidative profile

Footer: ↓—Decreased, ↑—Increased.

## Data Availability

No new data were created or analyzed in this study.

## References

[1] GBD 2021 Osteoarthritis Collaborators (2023). Global, Regional, and National Burden of Osteoarthritis, 1990–2020 and Projections to 2050: A Systematic Analysis for the Global Burden of Disease Study 2021. Lancet Rheumatol..

[2] Aghamohammadi D., Dolatkhah N., Bakhtiari F., Eslamian F., Hashemian M. (2020). Nutraceutical Supplements in Management of Pain and Disability in Osteoarthritis: A Systematic Review and Meta-Analysis of Randomized Clinical Trials. Sci. Rep..

[3] Liu C.-Y., Duan Y.-S., Zhou H., Wang Y., Tu J.-F., Bao X.-Y., Yang J.-W., Lee M.S., Wang L.-Q. (2024). Clinical Effect and Contributing Factors of Acupuncture for Knee Osteoarthritis: A Systematic Review and Pairwise and Exploratory Network Meta-Analysis. BMJ Evid.-Based Med..

[4] Prego-Dominguez J., Hadrya F., Takkouche B. (2016). Polyunsaturated Fatty Acids and Chronic Pain: A Systematic Review and Meta-Analysis. Pain Physician.

[5] Wang Y., Cao Z., Gao Y., Shao P., Gao S., Dong M., Tian Z., Feng Y., Xu J., Xiang C. (2025). Nutritional Interventions for Osteoarthritis: Targeting the Metabolism-Inflammation-Oxidative Stress Axis—Clinical Evidence and Translational Practice. Front. Nutr..

[6] Loeser R.F., Goldring S.R., Scanzello C.R., Goldring M.B. (2012). Osteoarthritis: A disease of the joint as an organ. Arthritis Rheum..

[7] Chen D., Shen J., Zhao W., Wang T., Han L., Hamilton J.L., Im H.J. (2017). Osteoarthritis: Toward a comprehensive understanding of pathological mechanism. Bone Res..

[8] Stewart H.L., Kawcak C.E. (2018). The Importance of Subchondral Bone in the Pathophysiology of Osteoarthritis. Front. Vet. Sci..

[9] De Roover A., Escribano-Núñez A., Monteagudo S., Lories R. (2023). Fundamentals of Osteoarthritis: Inflammatory Mediators in Osteoarthritis. Osteoarthr. Cartil..

[10] Gress K., Charipova K., An D., Hasoon J., Kaye A.D., Paladini A., Varrassi G., Viswanath O., Abd-Elsayed A., Urits I. (2020). Treatment recommendations for chronic knee osteoarthritis. Best Pr. Res. Clin. Anaesthesiol..

[11] Martinez-Moreno D. (2019). Cartilage Biomechanics: A Key Factor for Osteoarthritis Regenerative Medicine. Biochim. Biophys. Acta (BBA)—Mol. Basis Dis..

[12] Goldring M.B., Otero M. (2011). Inflammation in osteoarthritis. Curr. Opin. Rheumatol..

[13] Molnar V., Matišić V., Kodvanj I. (2021). Cytokines and chemokines involved in osteoarthritis pathogenesis. Int. J. Mol. Sci..

[14] Xue J., Wang J., Liu Q. (2013). Tumor necrosis factor-α induces ADAMTS-4 expression in human osteoarthritis chondrocytes. Mol. Med. Rep..

[15] Kapoor M., Martel-Pelletier J., Lajeunesse D. (2011). Role of proinflammatory cytokines in the pathophysiology of osteoarthritis. Nat Rev. Rheumatol..

[16] (2022). Surgical Management of Osteoarthritis of the Knee: Evidence-Based Clinical Practice Guideline.

[17] Sokolove J., Lepus C.M. (2013). Role of inflammation in the pathogenesis of osteoarthritis: Latest findings and interpretations. Ther. Adv. Musculoskelet. Dis..

[18] Zhang H., Cai D., Bai X. (2020). Macrophages regulate the progression of osteoarthritis. Osteoarthr. Cartil..

[19] Zeng N., Yan Z.P., Chen X.Y., Ni G.X. (2020). Infrapatellar Fat Pad and Knee Osteoarthritis. Aging Dis.

[20] Eymard F., Chevalier X. (2016). Inflammation of the infrapatellar fat pad. Jt. Bone Spine.

[21] Chang J., Liao Z., Lu M., Meng T., Han W., Ding C. (2018). Systemic and local adipose tissue in knee osteoarthritis. Osteoarthr. Cartil..

[22] Hwang H.S., Kim H.A. (2015). Chondrocyte Apoptosis in the Pathogenesis of Osteoarthritis. Int. J. Mol. Sci..

[23] Guo P., Alhaskawi A., Moqbel S.A.A., Pan Z. (2025). Recent development of mitochondrial metabolism and dysfunction in osteoarthritis. Front. Pharmacol..

[24] Dickson B.M., Roelofs A.J., Rochford J.J., Wilson H.M., De Bari C. (2019). The burden of metabolic syndrome on osteoarthritic joints. Arthritis Res. Ther..

[25] Wang T., He C. (2018). Pro-inflammatory cytokines: The link between obesity and osteoarthritis. Cytokine Growth Factor Rev..

[26] Kanthawang T., Bodden J., Joseph G.B. (2021). Obese and overweight individuals have greater knee synovial inflammation and associated structural and cartilage compositional degeneration: Data from the osteoarthritis initiative. Skelet. Radiol..

[27] Yoshimura N., Muraki S., Oka H. (2012). Accumulation of metabolic risk factors such as overweight, hypertension, dyslipidaemia, and impaired glucose tolerance raises the risk of occurrence and progression of knee osteoarthritis: A 3-year follow-up of the ROAD study. Osteoarthr. Cartil..

[28] Boden G. (2008). Obesity and free fatty acids. Endocrinol. Metab. Clin. N. Am..

[29] Lippiello L., Walsh T., Fienhold M. (1991). The association of lipid abnormalities with tissue pathology in human osteoarthritic articular cartilage. Metabolism.

[30] Datta P., Zhang Y., Parousis A., Sharma A., Rossomacha E., Endisha H., Wu B., Kacprzak I., Mahomed N.N., Gandhi R. (2017). High-fat diet-induced acceleration of osteoarthritis is associated with a distinct and sustained plasma metabolite signature. Sci. Rep..

[31] Tan L., Harper L.R., Armstrong A., Carlson C.S., Yammani R.R. (2021). Dietary Saturated Fatty Acid Palmitate Promotes Cartilage Lesions and Activates the Unfolded Protein Response Pathway in Mouse Knee Joints. PLoS ONE.

[32] Miao H., Chen L., Hao L., Zhang X., Chen Y., Ruan Z., Liang H. (2015). Stearic acid induces proinflammatory cytokine production partly through activation of lactate-HIF1α pathway in chondrocytes. Sci. Rep..

[33] Bautista A., Lee J., Delfino S., LaPreze D., Abd-Elsayed A. (2024). The Impact of Nutrition on Pain: A Narrative Review of Recent Literature. Curr. Pain Headache Rep..

[34] Franchini C., Biasini B., Sogari G., Wongprawmas R., Andreani G., Dolgopolova I., Gómez M.I., Roosen J., Menozzi D., Mora C. (2024). Adherence to the Mediterranean Diet and its association with sustainable dietary behaviors, sociodemographic factors, and lifestyle: A cross-sectional study in US University students. Nutr. J..

[35] Pitaraki E.E. (2017). The role of Mediterranean diet and its components on the progress of osteoarthritis. J. Frailty Sarcopenia Falls.

[36] Dyer J., Davison G., Marcora S.M., Mauger A.R. (2017). Effect of a Mediterranean Type Diet on Inflammatory and Cartilage Degradation Biomarkers in Patients with Osteoarthritis. J. Nutr. Health Aging.

[37] Veronese N., Koyanagi A., Stubbs B., Cooper C., Guglielmi G., Rizzoli R., Punzi L., Rogoli D., Caruso M.G., Rotolo O. (2019). Mediterranean diet and knee osteoarthritis outcomes: A longitudinal cohort study. Clin. Nutr..

[38] Malesza I.J., Malesza M., Walkowiak J., Mussin N., Walkowiak D., Aringazina R., Bartkowiak-Wieczorek J., Mądry E. (2021). High-Fat, Western-Style Diet, Systemic Inflammation, and Gut Microbiota: A Narrative Review. Cells.

[39] Collins K.H., Paul H.A., Reimer R.A., Seerattan R.A., Hart D.A., Herzog W. (2015). Relationship between inflammation, the gut microbiota, and metabolic osteoarthritis development: Studies in a rat model. Osteoarthr. Cartil..

[40] Berenbaum F., Griffin T.M., Liu-Bryan R. (2017). Review: Metabolic Regulation of Inflammation in Osteoarthritis. Arthritis Rheumatol..

[41] Lambova S.N., Batsalova T., Moten D., Stoyanova S., Georgieva E., Belenska-Todorova L., Kolchakova D., Dzhambazov B. (2021). Serum Leptin and Resistin Levels in Knee Osteoarthritis-Clinical and Radiologic Links: Towards Precise Definition of Metabolic Type Knee Osteoarthritis. Biomedicines.

[42] Mooney R.A., Sampson E.R., Lerea J., Rosier R.N., Zuscik M.J. (2011). High-fat diet accelerates progression of osteoarthritis after meniscal/ligamentous injury. Arthritis Res. Ther..

[43] Sidhu S.R.K., Kok C.W., Kunasegaran T., Ramadas A. (2023). Effect of Plant-Based Diets on Gut Microbiota: A Systematic Review of Interventional Studies. Nutrients.

[44] Ansari M.Y., Ahmad N., Haqqi T.M. (2020). Oxidative stress and inflammation in osteoarthritis pathogenesis: Role of polyphenols. Biomed. Pharmacother..

[45] Amirkhizi F., Ghoreishy S.M., Hamedi-Shahraki S., Asghari S. (2022). Higher dietary phytochemical index is associated with lower odds of knee osteoarthritis. Sci. Rep..

[46] Lv X., Deng X., Lai R., Liu S., Zou Z., Dai X., Luo Y., Yuan Q., Li Y. (2025). The association between dietary fiber intake and osteoarthritis: A cross-sectional study from the 1999–2018 U.S. National Health and Nutrition Examination Survey. J. Orthop. Surg. Res..

[47] Sirše M. (2022). Effect of Dietary Polyphenols on Osteoarthritis-Molecular Mechanisms. Life.

[48] Buhrmann C., Brockmueller A., Mueller A.L., Shayan P., Shakibaei M. (2021). Curcumin Attenuates Environment-Derived Osteoarthritis by Sox9/NF-kB Signaling Axis. Int. J. Mol. Sci..

[49] Buhrmann C., Popper B., Kunnumakkara A.B., Aggarwal B.B., Shakibaei M. (2019). Evidence That Calebin A, a Component of Curcuma Longa Suppresses NF-κB Mediated Proliferation, Invasion and Metastasis of Human Colorectal Cancer Induced by TNF-β (Lymphotoxin). Nutrients.

[50] Funk J.L., Frye J.B., Oyarzo J.N., Kuscuoglu N., Wilson J., McCaffrey G., Stafford G., Chen G., Lantz R.C., Jolad S.D. (2006). Efficacy and mechanism of action of turmeric supplements in the treatment of experimental arthritis. Arthritis Rheum..

[51] Shakibaei M., John T., Schulze-Tanzil G., Lehmann I., Mobasheri A. (2007). Suppression of NF-kappaB activation by curcumin leads to inhibition of expression of cyclo-oxygenase-2 and matrix metalloproteinase-9 in human articular chondrocytes: Implications for the treatment of osteoarthritis. Biochem. Pharmacol..

[52] Zimmermann-Klemd A.M., Reinhardt J.K., Nilsu T., Morath A., Falanga C.M., Schamel W.W., Huber R., Hamburger M., Gründemann C. (2020). Boswellia Carteri Extract and 3-O-Acetyl-Alpha-Boswellic Acid Suppress T Cell Function. Fitoterapia.

[53] Kimmatkar N., Thawani V., Hingorani L., Khiyani R. (2003). Efficacy and tolerability of Boswellia serrata extract in treatment of osteoarthritis of knee—A randomized double blind placebo controlled trial. Phytomedicine.

[54] Gupta P.K., Samarakoon S.M., Chandola H.M., Ravishankar B. (2011). Clinical evaluation of Boswellia serrata (Shallaki) resin in the management of Sandhivata (osteoarthritis). Ayu.

[55] Choi Y.-J., Jung J.I., Bae J., Lee J.K., Kim E.J. (2024). Evaluating the Anti-Osteoarthritis Potential of Standardized Boswellia serrata Gum Resin Extract in Alleviating Knee Joint Pathology and Inflammation in Osteoarthritis-Induced Models. Int. J. Mol. Sci..

[56] Luk H.Y., Appell C., Chyu M.C., Chen C.H., Wang C.Y., Yang R.S., Shen C.L. (2020). Impacts of Green Tea on Joint and Skeletal Muscle Health: Prospects of Translational Nutrition. Antioxidants.

[57] Ahmed S., Wang N., Lalonde M., Goldberg V.M., Haqqi T.M. (2004). Green Tea Polyphenol Epigallocatechin-3-Gallate (EGCG) Differentially Inhibits Interleukin-1β-Induced Expression of Matrix Metalloproteinase-1 and-13 in Human Chondrocytes. J. Pharmacol. Exp. Ther..

[58] Singh R., Ahmed S., Islam N., Goldberg V.M., Haqqi T.M. (2002). Epigallocatechin-3-gallate inhibits interleukin-1beta-induced expression of nitric oxide synthase and production of nitric oxide in human chondrocytes: Suppression of nuclear factor kappaB activation by degradation of the inhibitor of nuclear factor kappaB. Arthritis Rheum..

[59] Huang H.T., Cheng T.L., Ho C.J., Huang H.H., Lu C.C., Chuang S.C., Li J.Y., Lee T.C., Chen S.T., Lin Y.S. (2020). Intra-Articular Injection of (-)-Epigallocatechin 3-Gallate to Attenuate Articular Cartilage Degeneration by Enhancing Autophagy in a Post-Traumatic Osteoarthritis Rat Model. Antioxidants.

[60] Aziz D.M., Wsoo M.A., Ibrahim B.M. (2015). Antimicrobial and antioxidant activities of extracts from medicinal plant ginger (*Zingiber officinale*) and identification of components by gas chromatography. Afr. J. Plant Sci..

[61] Baskar V., Selvakumar K., Madhan R., Srinivasan G., Muralidharan M. (2012). Study on improving bioavailability ratio of anti-inflammatory compound from ginger through nano transdermal delivery. Asian J. Pharm. Clin. Res..

[62] Ma Y., Sun C., Wang Z., Chen C. (2025). Cartilage extracellular matrix regeneration with 10-gingerol via KEAP1-NRF2-ARE axis for osteoarthritis therapy. Arch. Pharmacal Res..

[63] Wang W., Sun L., Zhang P., Song J., Liu W. (2014). An anti-inflammatorycell-free collagen/resveratrol scaffold for repairing osteochondral defects in rabbits. Acta Biomater..

[64] Li W., Hu S., Chen X., Shi J. (2021). The Antioxidant Resveratrol Protects against Chondrocyte Apoptosis by Regulating the COX-2/NF-κB Pathway in Created Temporomandibular Osteoarthritis. Biomed. Res. Int..

[65] Hecht J.T., Veerisetty A.C., Wu J., Coustry F., Hossain M.G., Chiu F., Gannon F.H., Posey K.L. (2021). Primary Osteoarthritis Early Joint Degeneration Induced by Endoplasmic Reticulum Stress Is Mitigated by Resveratrol. Am. J. Pathol..

[66] Colletti A., Cicero A.F.G. (2021). Nutraceutical Approach to Chronic Osteoarthritis: From Molecular Research to Clinical Evidence. Int. J. Mol. Sci..

[67] Gambari L., Cellamare A., Grassi F., Grigolo B., Panciera A., Ruffilli A., Faldini C., Desando G. (2022). Overview of Anti-Inflammatory and Anti-Nociceptive Effects of Polyphenols to Halt Osteoarthritis: From Preclinical Studies to New Clinical Insights. Int. J. Mol. Sci..

[68] Ofosu F.K., Daliri E.B.M., Elahi F., Chelliah R., Lee B.H., Oh D.H. (2020). New Insights on the Use of Polyphenols as Natural Preservatives and Their Emerging Safety Concerns. Front. Sustain. Food Syst..

[69] Siddiqui R.A., Moghadasian M.H. (2020). Nutraceuticals and Nutrition Supplements: Challenges and Opportunities. Nutrients.

[70] Hu Y., Gui Z., Zhou Y., Xia L., Lin K., Xu Y. (2019). Quercetin alleviates rat osteoarthritis by inhibiting inflammation and apoptosis of chondrocytes, modulating synovial macrophages polarization to M2 macrophages. Free Radic. Biol. Med..

[71] Wang H., Yan Y., Pathak J.L., Hong W., Zeng J., Qian D., Hao B., Li H., Gu J., Jaspers R.T. (2023). Quercetin prevents osteoarthritis progression possibly via regulation of local and systemic inflammatory cascades. J. Cell. Mol. Med..

[72] Chen A.Y., Chen Y.C. (2013). A review of the dietary flavonoid, kaempferol on human health and cancer chemoprevention. Food Chem..

[73] Huang X., Pan Q., Mao Z., Wang P., Zhang R., Ma X., Chen J., You H. (2018). Kaempferol inhibits interleukin-1β stimulated matrix metalloproteinases by suppressing the MAPK-associated ERK and P38 signaling pathways. Mol. Med. Rep..

[74] Scandiffio R., Geddo F., Cottone E., Querio G., Antoniotti S., Gallo M.P., Maffei M.E., Bovolin P. (2020). Protective Effects of (E)-β-Caryophyllene (BCP) in Chronic Inflammation. Nutrients.

[75] Farì G., Megna M., Scacco S., Ranieri M., Raele M.V., Noya E.C., Macchiarola D., Bianchi F.P., Carati D., Gnoni A. (2023). Effects of Terpenes on the Osteoarthritis Cytokine Profile by Modulation of IL-6: Double Face versus Dark Knight?. Biology.

[76] Mattiuzzo E., Faggian A., Venerando R., Benetti A., Belluzzi E., Abatangelo G., Ruggieri P., Brun P. (2021). In Vitro Effects of Low Doses of β-Caryophyllene, Ascorbic Acid and d-Glucosamine on Human Chondrocyte Viability and Inflammation. Pharmaceuticals.

[77] Carvalho A.M.S., Heimfarth L., Santos K.A., Guimarães A.G., Picot L., Almeida J.R.G.S., Quintans J.S.S., Quintans-Júnior L.J. (2019). Terpenes as possible drugs for the mitigation of arthritic symptoms—A systematic review. Phytomedicine.

[78] Kabir M.T., Rahman M.H., Shah M., Jamiruddin M.R., Basak D., Al-Harrasi A., Bhatia S., Ashraf G.M., Najda A., El-Kott A.F. (2022). Therapeutic promise of carotenoids as antioxidants and anti-inflammatory agents in neurodegenerative disorders. Biomed. Pharmacother..

[79] Vafaei S., Wu X., Tu J., Nematollahi-Mahani S.N. (2022). The Effects of Crocin on Bone and Cartilage Diseases. Front. Pharmacol..

[80] Lei M., Guo C., Hua L., Xue S., Yu D., Zhang C., Wang D. (2017). Crocin Attenuates Joint Pain and Muscle Dysfunction in Osteoarthritis Rat. Inflammation.

[81] Ding Q., Zhong H., Qi Y., Cheng Y., Li W., Yan S., Wang X. (2013). Anti-arthritic effects of crocin in interleukin-1β-treated articular chondrocytes and cartilage in a rabbit osteoarthritic model. Inflamm. Res..

[82] Zhu B., Li G., Wu K., Luo Q., Wu X. (2025). Relationship between serum carotenoids and osteoarthritis or degenerative arthritis: A cross-sectional study using the National Health and Nutrition Examination Survey. Nutr. J..

[83] Zhou Y., Ming J., Deng M., Li Y., Li B., Li J., Ma Y., Chen Z., Wang G., Liu S. (2020). Chemically modified curcumin (CMC2.24) alleviates osteoarthritis progression by restoring cartilage homeostasis and inhibiting chondrocyte apoptosis via the NF-κB/HIF-2α axis. J. Mol. Med..

[84] Yabas M., Orhan C., Er B., Tuzcu M., Durmus A.S., Ozercan I.H., Sahin N., Bhanuse P., Morde A.A., Padigaru M. (2021). A Next Generation Formulation of Curcumin Ameliorates Experimentally Induced Osteoarthritis in Rats via Regulation of Inflammatory Mediators. Front. Immunol..

[85] Kann B., Spengler C., Coradini K., Rigo L.A., Bennink M.L., Jacobs K., Offerhaus H.L., Beck R.C., Windbergs M. (2016). Intracellular Delivery of Poorly Soluble Polyphenols: Elucidating the Interplay of Self-Assembling Nanocarriers and Human Chondrocytes. Anal. Chem..

[86] Yakubu J., Pandey A.V. (2024). Innovative Delivery Systems for Curcumin: Exploring Nanosized and Conventional Formulations. Pharmaceutics.

[87] Gupte P.A., Giramkar S.A., Harke S.M., Kulkarni S.K., Deshmukh A.P., Hingorani L.L., Mahajan M.P., Bhalerao S.S. (2019). Evaluation of the Efficacy and Safety of Capsule Longvida^®^ Optimized Curcumin (Solid Lipid Curcumin Particles) in Knee Osteoarthritis: A Pilot Clinical Study. J. Inflamm. Res..

[88] Clinton C.M., O’Brien S., Law J., Renier C.M., Wendt M.R. (2015). Whole-Foods, Plant-Based Diet Alleviates the Symptoms of Osteoarthritis. Arthritis.

[89] Peck J., Slovek A., Miro P., Vij N., Traube B., Lee C., Berger A.A., Kassem H., Kaye A.D., Sherman W.F. (2021). A Comprehensive Review of Viscosupplementation in Osteoarthritis of the Knee. Orthop. Rev..

[90] Kong H., He Q., Han J., Zhang X.-A. (2025). Nanomaterial-Based Drug Delivery Systems Targeting Functional Cells for Osteoarthritis Treatment: Mechanisms, Challenges and Future Prospects. Int. J. Nanomed..

[91] Guo Z., Lin J., Sun K., Guo J., Yao X., Wang G., Hou L., Xu J., Guo J., Guo F. (2022). Deferoxamine Alleviates Osteoarthritis by Inhibiting Chondrocyte Ferroptosis and Activating the Nrf2 Pathway. Front. Pharmacol..

[92] Liu-Bryan R. (2015). Inflammation and intracellular metabolism: New targets in OA. Osteoarthr. Cartil..

[93] Liu X., Machado G.C., Eyles J.P., Ravi V., Hunter D.J. (2017). Dietary Supplements for Treating Osteoarthritis: A Systematic Review and Meta-Analysis. Br. J. Sports Med..

[94] Paultre K., Cade W., Hernandez D., Reynolds J., Greif D., Best T.M. (2021). Therapeutic Effects of Turmeric or Curcumin Extract on Pain and Function for Individuals with Knee Osteoarthritis: A Systematic Review. BMJ Open Sport Exerc. Med..

[95] Asadi S., Grafenauer S., Burley C.V., Fitzgerald C., Humburg P., Parmenter B.J. (2025). The Effectiveness of Dietary Intervention in Osteoarthritis Management: A Systematic Review and Meta-Analysis of Randomized Clinical Trials. Eur. J. Clin. Nutr..

[96] Navarro S.L., White E., Kantor E.D., Zhang Y., Rho J., Song X., Milne G.L., Lampe P.D., Lampe J.W. (2015). Randomized Trial of Glucosamine and Chondroitin Supplementation on Inflammation and Oxidative Stress Biomarkers and Plasma Proteomics Profiles in Healthy Humans. PLoS ONE.

[97] Valsamidou E., Amerikanou C., Tzavara C., Skarpas G., Mariolis-Sapsakos T.D., Zoumpoulakis P., Kaliora A.C. (2023). A Standardized Nutraceutical Supplement Contributes to Pain Relief, Improves Quality of Life and Regulates Inflammation in Knee Osteoarthritis Patients; A Randomized Clinical Trial. Heliyon.

[98] Wang J., Wang X., Cao Y., Huang T., Song D., Tao H. (2018). Therapeutic potential of hyaluronic acid/chitosan nanoparticles for the delivery of curcuminoid in knee osteoarthritis and an in vitro evaluation in chondrocytes. Int. J. Mol. Med..

[99] Wu R., Guo Y., Chen Y., Zhang J. (2025). Osteoarthritis burden and inequality from 1990 to 2021: A systematic analysis for the global burden of disease Study 2021. Sci. Rep..

[100] Zeng J., Franklin D.K., Das A., Hirani V. (2023). The effects of dietary patterns and food groups on symptomatic osteoarthritis: A systematic review. Nutr. Diet..

[101] Morales-Ivorra I., Romera-Baures M., Roman-Viñas B., Serra-Majem L. (2018). Osteoarthritis and the Mediterranean Diet: A Systematic Review. Nutrients.

[102] Halegoua-DeMarzio D., Navarro V., Ahmad J., Avula B., Barnhart H., Barritt A.S., Bonkovsky H.L., Fontana R.J., Ghabril M.S., Hoofnagle J.H. (2023). Liver Injury Associated with Turmeric—A Growing Problem: Ten Cases from the Drug-Induced Liver Injury Network [DILIN]. Am. J. Med..

[103] Likhitsup A., Chen V.L., Fontana R.J. (2024). Estimated Exposure to 6 Potentially Hepatotoxic Botanicals in US Adults. JAMA Netw. Open.

[104] Grajecki D., Ogica A., Boenisch O., Hübener P., Kluge S. (2022). Green tea extract-associated acute liver injury: Case report and review. Clin. Liver Dis..

[105] Kolasinski S.L., Neogi T., Hochberg M.C., Oatis C., Guyatt G., Block J., Callahan L., Copenhaver C., Dodge C., Felson D. (2020). 2019 American College of Rheumatology/Arthritis Foundation Guideline for the Management of Osteoarthritis of the Hand, Hip, and Knee. Arthritis Rheum..

[106] Sato B.Y.S., Chong S.I., Souza N.M.P., Lazo R.E.L., Pontarolo R., Rego F.G.M., Ferreira L.M., Sari M.H.M. (2025). Nanocarriers Containing Curcumin and Derivatives for Arthritis Treatment: Mapping the Evidence in a Scoping Review. Pharmaceutics.

[107] Gambaro F.M., Ummarino A., Torres Andón F., Ronzoni F., Di Matteo B., Kon E. (2021). Drug Delivery Systems for the Treatment of Knee Osteoarthritis: A Systematic Review of In Vivo Studies. Int. J. Mol. Sci..

[108] Deng W., Wang T., Li L., Xiao X., Xu Y., Li Q., Zhou Q., Yin Y., Yang H., Gong K. (2025). A review of nanomaterials in osteoarthritis treatment and immune modulation. Regen. Biomater..

